# Inflammatory Polymorphisms (IL-6 *rs1800796*, IL-10 *rs1800896*, TNF-α *rs1800629*, and IFITM3 *rs12252*) Are Not Associated with Post-COVID Symptoms in Previously Hospitalized COVID-19 Survivors

**DOI:** 10.3390/v16020275

**Published:** 2024-02-09

**Authors:** César Fernández-de-las-Peñas, Gema Díaz-Gil, Antonio Gil-Crujera, Stella M. Gómez-Sánchez, Silvia Ambite-Quesada, Juan Torres-Macho, Pablo Ryan-Murua, Ana I. Franco-Moreno, Oscar J. Pellicer-Valero, Lars Arendt-Nielsen, Rocco Giordano

**Affiliations:** 1 Department of Physical Therapy, Occupational Therapy, Rehabilitation and Physical Medicine, Universidad Rey Juan Carlos, 28922 Alcorcón, Spain; silvia.ambite.quesada@urjc.es; 2 Center for Neuroplasticity and Pain (CNAP), Sensory Motor Interaction (SMI), Department of Health Science and Technology, Faculty of Medicine, Aalborg University, DK-9220 Aalborg, Denmark; lan@hst.aau.dk (L.A.-N.); rg@hst.aau.dk (R.G.); 3 Research group GAMDES, Department of Basic Health Sciences, Universidad Rey Juan Carlos (URJC), 28933 Madrid, Spain; gema.diaz@urjc.es (G.D.-G.); antonio.gil@urjc.es (A.G.-C.); stella.gomez@urjc.es (S.M.G.-S.); 4 Department of Internal Medicine, Hospital Universitario Infanta Leonor-Virgen de la Torre, 28031 Madrid, Spain; juan.torresm@salud.madrid.org (J.T.-M.); pabloryan@gmail.com (P.R.-M.); anaisabel.franco@salud.madrid.org (A.I.F.-M.); 5 Department of Medicine, School of Medicine, Universidad Complutense de Madrid, 28040 Madrid, Spain; 6 Image Processing Laboratory (IPL), Universitat de València, Parc Científic, Paterna, 46100 València, Spain; oscar.pellicer@uv.es; 7 Department of Gastroenterology & Hepatology, Mech-Sense, Clinical Institute, Aalborg University Hospital, DK-9000 Aalborg, Denmark; 8 Steno Diabetes Center North Denmark, Clinical Institute, Aalborg University Hospital, DK-9000 Aalborg, Denmark; 9 Department of Oral and Maxillofacial Surgery, Aalborg University Hospital, DK-9000 Aalborg, Denmark

**Keywords:** single-nucleotide polymorphism, IL-6, IL-10, TNF-α, IFITM3, genotypes, post-COVID

## Abstract

The aim of this study was to identify the association between four selected inflammatory polymorphisms with the development of long-term post-COVID symptoms in subjects who had been hospitalized due to SARS-CoV-2 infection during the first wave of the pandemic. These polymorphisms were selected as they are associated with severe COVID-19 disease and cytokine storm, so they could be important to prognoses post-COVID. A total of 408 (48.5% female, age: 58.5 ± 14.0 years) previously hospitalized COVID-19 survivors participated. The three potential genotypes of the following four single-nucleotide polymorphisms, IL-6 *rs1800796*, IL-10 *rs1800896*, TNF-α *rs1800629*, and IFITM3 *rs12252*, were obtained from non-stimulated saliva samples of the participants. The participants were asked to self-report the presence of any post-COVID symptoms (defined as symptoms that had started no later than one month after SARS-CoV-2 acute infection) and whether the symptoms persisted at the time of the study. At the time of the study (mean: 15.6, SD: 5.6 months after discharge), 89.4% of patients reported at least one post-COVID symptom (mean number of symptoms: 3.0; SD: 1.7). Fatigue (69.3%), pain (40.9%), and memory loss (27.2%) were the most prevalent post-COVID symptoms in the total sample. Overall, no differences in the post-COVID symptoms depending on the IL-6 *rs1800796*, IL-10 *rs1800896*, TNF-α *rs1800629*, and IFITM3 *rs12252* genotypes were seen. The four SNPs assessed, albeit having been previously associated with inflammation and COVID-19 severity, did not cause a predisposition to the development of post-COVID symptoms in the previously hospitalized COVID-19 survivors.

## 1. Introduction

The identification of the severe acute respiratory syndrome coronavirus 2 (SARS-CoV-2) virus, the agent responsible of the worldwide spread of coronavirus disease 2019 (COVID-19), was possible due to the analysis of samples from the lower respiratory tracts of infected individuals [1]. Several studies have focused on the specific viral mechanisms of SARS-CoV-2 infection, e.g., the entry pathways via different receptors (e.g., surface receptor for S1 of the angiotensin-converting enzyme 2 —ACE2—or transmembrane protease serine-2—TMPRSS2) [2] or the underlying mechanisms behind the pro-inflammatory response (i.e., cytokine storm) [3]. In fact, different types of cytokines play an important role in the pathophysiology of acute COVID-19. For instance, interleukin 6 (IL-6) and interleukin 10 (IL-10) exhibit a clear overproduction during an acute SARS-CoV-2 infection, particularly in patients with severe illness [4]. Thus, tumor necrosis factor-α (TNF-α) and interferon-induced transmembrane (IFITM) proteins have also been associated with severe COVID-19 and higher inflammatory states [5].

Different polymorphisms can account for the variability in expression of these pro-inflammatory cytokines [6]. For instance, genotyping of cytokine-related single-nucleotide polymorphisms (SNPs), e.g., IL-6 *rs1800796* [7], IL-10 *rs1800896* [8], TNF-α *rs1800629* [9], and IFITM3 *rs12252* [10], has shown them to underlie the differential viral virulence and severity of COVID-19. Looking at the research, most studies have investigated the potential role of SNPs at the acute phase of SARS-CoV-2 infection, focusing on the risk of developing the severe form of the COVID-19 illness [6,7,8,9,10]. Our understanding of the role of these SNPs in symptoms persisting after the acute phase of SARS-CoV-2 infection is still in its infancy.

A growing healthcare problem starting from the beginning of the COVID-19 outbreak has been the presence of long-lasting symptoms once the acute phase of SARS-CoV-2 infection has passed. The presence of symptoms after the acute phase of a SARS-CoV-2 infection is called long COVID [11] or post-COVID-19 condition [12]. The World Health Organization (WHO) adopted the term post-COVID-19 condition as defined by Soriano et al. [12]: “post-COVID-19 condition occurs in people with a history of probable or confirmed SARS-CoV-2 infection, usually three months from the onset of infection, with symptoms that last for at least two months and cannot be explained by an alternative medical diagnosis. Common symptoms include, but are not limited to, fatigue, shortness of breath (dyspnea), and cognitive dysfunction, and generally have an impact on everyday functioning” [12]. Post-COVID-19 condition or long COVID is a heterogeneous condition, and patients can experience a plethora of symptoms affecting multiple systems [13]. In fact, more than 100 symptoms affecting the cardiovascular, neurological, immune, respiratory, musculoskeletal, and gastrointestinal systems have been attributed to SARS-CoV-2 since the beginning of the pandemic [14]. With increasing evidence growing each week, and after three years of pandemic, recent meta-analyses have reported the presence of post-COVID symptoms one [15,16] or two [17] years after an acute SARS-CoV-2 infection in almost 25–30% of patients. Thus, the Global Burden of Disease Long COVID study (which included 1.2 million of subjects who had experienced an acute symptomatic SARS-CoV-2 infection) reported that 15% of COVID-19 survivors exhibit at least one post-COVID symptom one year after infection [18].

The underlying mechanisms explaining the presence of post-COVID symptoms are not completely understood, and different mechanisms have been proposed [19]. Long-lasting inflammation is proposed as a key mechanism contributing to the development of long COVID [20]. However, the evidence on the presence of inflammatory biomarkers in patients with post-COVID symptoms is still conflicting [21,22]. For instance, Lai et al. concluded that individuals with long COVID exhibit increased levels of 79 biomarkers but decreased levels of 29 biomarkers [21]. On the contrary, Williams et al. found reduced levels of IL-6, IL-2, IL-17, IL-13, and IL-4 in individuals with long COVID when compared with those without symptoms [22].

Due to the role of the inflammatory response in SARS-CoV-2 infection [23], it is possible that SNPs associated with the inflammatory response and associated with more severe illness can contribute to the development of long-lasting post-COVID symptoms. In a previous study, our research group observed that IL-6 *rs1800796*, IL-10 *rs1800896*, TNF-α *rs1800629*, and IFITM3 *rs12252* SNPs, which are usually associated with the inflammatory and immune response, were not associated with a higher risk of developing post-COVID pain [24]. No data on the association of these four inflammatory SNPs with the presence of long-term post-COVID symptoms are available. Therefore, the aim of the current study was to investigate the association between the IL-6 *rs1800796*, IL-10 *rs1800896*, TNF-α *rs1800629*, and IFITM3 *rs12252* polymorphisms and the presence of post-COVID symptoms in previously hospitalized COVID-19 survivors.

## 2. Methods

### 2.1. Participants

This study included individuals who had been hospitalized due to SARS-CoV-2 infection at four urban hospitals in Madrid (Spain) from March to May 2020 (first wave of the pandemic). The diagnosis of SARS-CoV-2 infection should have been confirmed using reverse transcription–polymerase chain reaction (RT–PCR) assay of a nasopharyngeal and oral swab sample, as well as clinical/radiological findings on hospital admission. Some of the participants in the current analysis were previously included in our previous study on pain [24], but the current data presented here are new, are based on a different sample, and have not previously been published. The study was approved by the Institutional Ethics Committees of all the institutions/hospitals involved (URJC0907202015920; HCSC20/495E, HSO25112020; HUFA 20/126; HUIL/092-20). All the participants provided their written informed consent prior to the collection of any data.

### 2.2. DNA Collection and Genotyping

The genotyping collection and management were the same as in our previous study and have been previously published [24]. Genotyping was obtained from unstimulated whole saliva samples collected from each subject, as previously described. The saliva samples were centrifuged at 3000 rpm for 15 min to obtain the cell sediment and stored at −20 °C until the analysis.

Genomic DNA was extracted from 500 mL of saliva using a MagMAX™ DNA Multi-Sample Ultra 2.0 Kit (Thermo Fisher Scientific Inc, Hemel Hempstead, Hertfordshire, UK). We extracted DNA using the KingFisher Flex purification robot (Thermo Fisher). The purity and concentration of the resulting DNA were assessed using Quant-iT™ PicoGreen™ dsDNA reagent (Thermo Fisher). The DNA was diluted to 5 ng/μL using 1× Tris-EDTA (TE) buffer (Sigma-Aldrich, Dorset, UK). The qPCR reaction mixtures of 10 μL contained a total of 10 ng of gDNA as a PCR template, 1× TaqMan Gene Expression PCR Master Mix, and 0.6× genotyping TaqMan probe assay [24].

A predesigned TaqMan^®^ SNP Genotyping Assay (Thermo Fisher Scientific Inc, Hertfordshire, UK) was used for genotyping the SNPs using a real-time PCR reaction (RT–PCR). The real-time PCR plates were run using the QuantStudio 12K Flex system (Thermo Fisher) of the Genomics Unit (Madrid Science Park Foundation, Spain) under the standard conditions (95° for 10 min and 40 two-step cycles consisting of 95 °C for 15 s and 60 °C for 1 min) and analyzed using the Genotyping app in Thermo Fisher Cloud. Identification of each of the possible variants of each SNP was conducted using specific fluorescent dyes [24].

Three possible genotypes (C/G, C/C, G/G) associated with the IL-6 *rs1800796* polymorphism were derived from a C→G substitution at the following sequence:

ATGGCCAGGCAGTTCTACAACAGCC [C/G] CTCACAGGGGAGCCAGAACACAGA.

Three possible genotypes (C/C, T/C, T/T) associated with the IL-10 *rs1800896* polymorphism were derived from a T→C substitution at the following sequence:

TCCTCTTACCTATCCCTACTTCCCC [T/C] TCCCAAAGAAGCCTTAGTAGTGTTG.

Three possible genotypes (A/A, A/G, G/G) related to the TNF-α *rs1800629* polymorphism were derived from an A→G substitution at the following sequence:

GAGGCAATAGGTTTTGAGGGGCATG [A/G] GGACGGGGTTCAGCCTCCAGGGTCC.

Three possible genotypes (A/A, A/G, G/G) associated with the IFITM3 *rs12252* polymorphism were derived from an A→G substitution at the following sequence:

GCATCTCATAGTTGGGGGGCTGGCC [A/G] CTGTTGACAGGAGAGAAGAAGGTTT.

### 2.3. Collection Data

The demographic (age, gender, height, weight), clinical (medical comorbidities), and hospitalization (intensive care unit (ICU) admission, days in hospital) data were collected from medical records.

Participants who agreed to participate were scheduled for a face-to-face appointment with a healthcare professional. They were asked to self-report the presence of symptoms that appeared after hospitalization due to SARS-CoV-2 infection (no later than one month after) and whether the symptom(s) persisted at the time of the study. A list of symptoms (e.g., fatigue, dyspnea, anosmia, ageusia, brain fog, hair loss, pain, or concentration loss) was systematically used, although participants were free to report any symptom that they suffered from.

### 2.4. Statistical Analysis

The data were collected using STATA 16.1 and processed using Python’s library pandas 0.25.3. Means and standard deviation (SD) are presented for quantitative data and the number of cases (percentages) are presented for categorical data. Chi-squared (χ2) tests were applied to assess the deviation in the genotype distribution from Hardy–Weinberg equilibrium. Differences in the prevalence of post-COVID symptoms by the genotype frequencies of each polymorphism were analyzed using χ2 tests. Thus, differences in the continuous variables by the genotype frequencies of each polymorphism were analyzed using one-way-ANOVA tests. The Shapiro–Wilk test was used to assess the assumption of normality. For all inferences, the level of significance was set a priori at 0.05, with the *p*-values from all tests being corrected (Holm–Bonferroni correction).

## 3. Results

A total of 450 Hispanic/Latin patients who were hospitalized due to SARS-CoV-2 infection from March to May 2020 were initially invited to participate during the period (September 2021 to February 2022). A total of 42 (9.3%) patients were excluded as follows: 1, refused to participate (n = 19); 2, previous autoimmune pathology (n = 10); 3, pregnancy (n = 5); and 4, saliva sample compromised during genotyping analyses (n = 8). Finally, completed data from 408 (48.5% female, age: 58.5 ± 14.0 years) COVID-19 survivors were obtained with a follow-up period of 15.6 (SD 5.6) months after hospital discharge. At the time of the study, 365 (89.4%) patients reported post-COVID symptoms (mean symptoms number: 3.0; SD: 1.7). Fatigue (69.3%), pain (40.9%), and memory loss (27.2%) were the most prevalent post-COVID symptoms in the total sample (Table 1).

The genotype distributions did not deviate from those expected based on the Hardy–Weinberg equilibrium.

Overall, no differences in the post-COVID symptoms depending on the IL-6 *rs1800796* (Table 2), IL-10 *rs1800896* (Table 3), TNF-α *rs1800629* (Table 4), and IFITM3 *rs12252* (Table 5) genotypes were observed. We only found that the A allele of the TNF-α *rs1800629* polymorphism and the G allele of the IFITM3 *rs12252* polymorphism were more prevalent in obese individuals (*p* < 0.01), although these associations were based on a small number of subjects (Table 4 and Table 5).

No sex differences in the distribution of the genotypes of the IL-6 *rs1800796* (*p* = 0.757, Table 2), IL-10 *rs1800896* (*p* = 0.372, Table 3), TNF-α *rs1800629* (*p* = 0.577, Table 4), and IFITM3 *rs12252* (*p* = 0.349, Table 5) polymorphisms were identified.

## 4. Discussion

There is evidence supporting the role of inflammation in the acute COVID-19 phase [4,5] and also in the post-COVID phase [20]. Thus, several polymorphisms associated with inflammation have been related to severe COVID-19 illness in previous studies [7,8,9,10]. Our study did not find an association between four SNPs associated with severe COVID-19, e.g., IL-6 *rs1800796*, IL-10 *rs1800896*, TNF-α *rs1800629*, and IFITM3 *rs12252*, and the presence of long-lasting post-COVID symptoms more than one year after infection.

We found that almost 90% of our sample of COVID-19 survivors exhibited at least one post-COVID symptom up to 18 months after discharge. Our prevalence rate is much higher than some meta-analyses reporting that 25–30% of COVID-19 survivors exhibit post-COVID symptoms one year after infection [15,16,18]. There are several potential explanations for the observed prevalence rate of post-COVID symptoms in our study. Firstly, the sample included patients who were infected during the initial wave of the pandemic. During this period, the historical SARS-CoV-2 strain was the predominant variant and known as the most aggressive. It has been observed that the prevalence of post-COVID symptoms tends to be higher in patients infected with the historical strain compared to those infected with later variants of concern, such as Alpha, Beta, Delta, or Omicron [25,26]. Secondly, all the participants in this study were infected and developed post-COVID symptoms before being vaccinated. The current evidence suggests that vaccination can decrease the risk of developing post-COVID symptoms if administered before infection, but its effects in patients with ongoing post-COVID symptomatology is unclear [27]. Third, our study included a cohort of hospitalized COVID-19 survivors and hence with moderate to severe COVID-19 illness. Although both hospitalized and non-hospitalized patients can develop post-COVID symptoms, hospitalized patients seem to exhibit a higher risk of some post-COVID symptoms, e.g., dyspnea, pain, and hair loss, than non-hospitalized patients [28]. Thus, we also observed that fatigue, pain, and memory loss were the most prevalent post-COVID symptoms, in agreement with previous meta-analyses [29,30,31].

The underlying mechanisms explaining the development of post-COVID symptoms are not fully understood, and different mechanisms are proposed: viral persistence, long-lasting inflammation, immune system dysregulation, autoimmunity, the reactivation of latent infections, endothelial dysfunction, and alteration in the gut microbiota [32]. Our study did not find an association between the four SNPs associated with inflammation and the development of post-COVID symptoms. The current results agree with a previous study showing that other polymorphisms associated with SARS-CoV-2 trophism, e.g., ACE2 *rs2285666*, ACE2 *rs2074192*, TMPRSS2 *rs12329760*, and TMPRSS2 *rs2070788*, did not predispose patients to developing post-COVID symptoms either [33]. The lack of a potential influence of inflammatory SNPs on post-COVID symptoms does not exclude the role of the products (inflammatory biomarkers levels) regulated by these polymorphisms. In fact, long-lasting systemic inflammation after the acute COVID-19 phase has been associated with a higher number of post-COVID symptoms [20]; however, it seems that the inflammatory biomarker levels are highly fluctuating [21,22]. Thus, it has been speculated that specific genes could influence particular post-COVID symptoms, although this hypothesis has still not been investigated. Finally, it is also possible that multiple genetic variants potentially modulate the inflammatory response [34].

Finally, the results of the current study should be considered according to its potential limitations. First, the sample consisted of previously hospitalized COVID-19 survivors; therefore, the role of the investigated polymorphisms in non-hospitalized patients should not be extrapolated. Thus, the cohort of this study consisted of patients infected during the first wave of the COVID-19 pandemic, when the historical SARS-CoV-2 variant was predominant. It remains unclear whether the investigated SNPs are associated with post-COVID symptoms in individuals infected with other SARS-CoV-2 variants of concern, although this seems unlikely. Second, the data were self-reported and collected longer than one year after hospitalization. Although we specifically asked for symptoms starting no later than one month after the infection, we cannot exclusively attribute to SARS-CoV-2 infection their development. Additionally, the present study focused solely on four polymorphisms commonly linked to COVID-19 severity and inflammation. At present, it is unknown whether analyses of different SNPs might produce different outcomes. Population-based studies that include whole-genome analysis could be instrumental in identifying additional genes associated with post-COVID symptoms.

## 5. Conclusions

This study showed that four polymorphisms associated with inflammation and severe COVID-19, e.g., IL-6 *rs1800796*, IL-10 *rs1800896*, TNF-α *rs1800629*, and IFITM3 *rs12252*, did not cause predisposition to developing post-COVID symptomatology in a cohort of previously hospitalized COVID-19 survivors infected during the first wave of the pandemic.

## Figures and Tables

**Table 1:** Pre-infection data and post-COVID symptoms of the total sample.

	Total Sample (n = 408)
Age, mean (SD), years	58.5 (14.0)
Sex, female n (%)	198 (48.5%)
Weight, mean (SD), kg.	80.1 (17.0)
Height, mean (SD), cm.	166.5 (9.5)
Number of co-morbidities, mean (SD)	1.2 (1.0)
Medical co-morbidities, n (%)	
Hypertension	143 (35.0%)
Obesity	98 (24.0%)
Diabetes	43 (10.5%)
Asthma	38 (9.3%)
Cardiovascular Diseases	38 (9.3%)
Chronic Obstructive Pulmonary Disease	10 (2.5%)
Rheumatological Diseases	3 (0.7%)
Number of post-COVID symptoms, mean (SD)	3.0 (1.7)
Post-COVID symptoms, n (%)	
Fatigue	283 (69.3%)
Pain Symptoms	167 (40.9%)
Memory Loss	111 (27.2%)
Hair Loss	105 (25.7%)
Concentration Loss	47 (11.5%)
Cognitive Blunting—Brain Fog	45 (11.0%)
Dyspnoea	80 (19.6%)
Ocular Disorders	45 (11.0%)
Skin Rashes	56 (13.7%)
Anosmia	39 (9.5%)
Gastrointestinal Disorders	29 (7.1%)
Ageusia	23 (5.6%)
Days in hospital, mean (SD)	8.2 (7.8)

**Table 2:** Pre-infection data and post-COVID symptoms according to the IL-6 *rs1800796* polymorphism.

	G/G (n = 322)	C/G (n = 78)	C/C (n = 8)	*p*-Value
Age, mean (SD), years	58.7 (14.0)	58.5 (14.5)	57.0 (14.5)	0.935
Sex, female n (%)	158 (49.1%)	35 (44.9%)	5 (62.5%)	0.757
Weight, mean (SD), kg.	80.1 (16.7)	79.7 (17.7)	76.2 (20.4)	0.806
Height, mean (SD), cm.	166.5 (10.0)	167 (8.8)	163 (8.8)	0.577
Number of co-morbidities, mean (SD)	1.2 (0.95)	1.3 (1.0)	0.75 (0.7)	0.310
Medical co-morbidities, n (%)				
Hypertension	110 (34.1%)	31 (39.7%)	2 (25.0%)	0.672
Obesity	78 (24.2%)	19 (24.4%)	1 (12.5%)	0.798
Diabetes	30 (9.3%)	13 (16.7%)	0 (0.0%)	0.131
Asthma	28 (8.7%)	10 (12.8%)	0 (0.0%)	0.385
Cardiovascular Diseases	30 (9.3%)	8 (10.25%)	0 (0.0%)	0.663
Chronic Obstructive Pulmonary Disease	10 (3.1%)	0 (0.0%)	0 (0.0%)	0.263
Rheumatological Diseases	3 (0.9%)	0 (0.0%)	0 (0.0%)	0.669
Number of post-COVID symptoms, mean (SD)	3.1 (1.7)	2.7 (1.7)	3.0 (1.8)	0.195
Post-COVID symptoms, n (%)				
Fatigue	228 (70.8%)	49 (62.8%)	6 (75.0%)	0.735
Pain Symptoms	135 (41.9%)	29 (37.2%)	3 (37.5%)	0.981
Memory Loss	89 (27.6%)	21 (26.9%)	1 (12.5%)	0.783
Hair Loss	86 (26.7%)	17 (21.8%)	2 (25.0%)	0.985
Concentration Loss	37 (11.5%)	10 (12.8%)	0 (0.0%)	0.595
Cognitive Blunting—Brain Fog	38 (11.8%)	7 (9.0%)	0 (0.0%)	0.507
Dyspnoea	69 (21.4%)	10 (12.8%)	1 (12.5%)	0.275
Ocular Disorders	37 (11.5%)	6 (7.7%)	2 (25.0%)	0.322
Anosmia	30 (9.3%)	8 (10.25%)	1 (12.5%)	0.936
Skin Rashes	41 (12.7%)	12 (15.4%)	3 (37.5%)	0.159
Gastrointestinal Disorders	22 (6.8%)	6 (7.7%)	1 (12.5%)	0.819
Ageusia	14 (4.3%)	8 (10.25%)	1 (12.5%)	0.102
Days in hospital, mean (SD)	8.3 (8.2)	7.1 (4.9)	7.0 (6.3)	0.422

**Table 3:** Pre-infection data and post-COVID symptoms according to the IL-10 *rs1800896* polymorphism.

	T/T (n = 163)	T/C (n = 183)	C/C (n = 62)	*p*-Value
Age, mean (SD), years	58.2 (14.2)	59.5 (13.9)	57.5 (13.5)	0.506
Sex, female n (%)	82 (50.3%)	93 (50.8%)	23 (37.1%)	0.372
Weight, mean (SD), kg.	81.5 (19.5)	78.5 (14.3)	80.5 (16.7)	0.225
Height, mean (SD), cm.	167 (9.5)	165.5 (9.5)	169 (10.0)	0.105
Number of co-morbidities, mean (SD)	1.2 (1.0)	1.3 (0.9)	1.0 (1.0)	0.160
Medical co-morbidities, n (%)				
Hypertension	60 (36.8%)	66 (36.1%)	17 (27.4%)	0.541
Obesity	41 (25.1%)	45 (24.6%)	12 (19.3%)	0.714
Diabetes	17 (10.4%)	21 (11.5%)	5 (8.1%)	0.773
Asthma	17 (10.4%)	17 (9.3%)	4 (6.4%)	0.683
Cardiovascular Diseases	13 (8.0%)	18 (9.8%)	7 (11.3%)	0.730
Chronic Obstructive Pulmonary Disease	7 (4.3%)	2 (1.1%)	1 (1.6%)	0.149
Rheumatological Diseases	0 (0.0%)	3 (1.6%)	0 (0.0%)	0.158
Number of post-COVID symptoms, mean (SD)	3.0 (1.6)	2.9 (1.7)	2.9 (1.9)	0.481
Post-COVID symptoms, n (%)				
Fatigue	116 (71.2%)	123(67.2%)	44 (71.0%)	0.895
Pain Symptoms	70 (42.9%)	74 (40.4%)	24 (38.7%)	0.895
Memory Loss	42 (25.8%)	51 (27.9%)	19 (30.4%)	0.814
Hair Loss	39 (23.9%)	51 (27.9%)	15 (24.2%)	0.742
Concentration Loss	18 (11.05%)	19 (10.4%)	10 (16.1%)	0.501
Cognitive Blunting—Brain Fog	10 (11.7%)	21 (11.5%)	5 (8.05%)	0.745
Dyspnoea	39 (23.9%)	27 (14.7%)	14 (22.6%)	0.134
Ocular Disorders	21 (12.9%)	17 (9.3%)	7 (11.3%)	0.602
Anosmia	12 (7.4%)	20 (10.9%)	7 (11.3%)	0.502
Skin Rashes	25 (15.3%)	26 (14.2%)	5 (8.05%)	0.409
Gastrointestinal Disorders	10 (6.1%)	14 (7.6%)	5 (8.05%)	0.831
Ageusia	6 (3.7%)	11 (6.0%)	6 (9.7%)	0.229
Days in hospital, mean (SD)	7.8 (6.0)	8.3 (9.1)	8.0 (7.2)	0.793

**Table 4:** Pre-infection data and post-COVID symptoms according to the TNF-α *rs1800629* polymorphism.

	G/G (n = 324)	A/G (n = 78)	A/A (n = 6)	*p*-Value
Age, mean (SD), years	58.4 (14.0)	60.0 (14.3)	55.7 (6.5)	0.580
Sex, female n (%)	153 (47.2%)	43 (55.1%)	2 (33.3%)	0.577
Weight, mean (SD), kg.	79.5 (16.5)	80.0 (18.0)	101.2 (12.2)	0.008
Height, mean (SD), cm.	166.5 (9.5)	166 (9.5)	177.5 (14.7)	0.02
Number of co-morbidities, mean (SD)	1.25 (1.0)	1.1 (1.0)	1.5 (0.85)	0.403
Medical co-morbidities, n (%)				
Hypertension	114 (35.1%)	28 (35.9%)	1 (16.7%)	0.742
Obesity	78 (24.1%)	15 (19.25%)	5 (83.3%)	0.008
Diabetes	35 (10.8%)	7 (9.0%)	1 (16.7%)	0.812
Asthma	32 (9.9%)	5 (6.4%)	1 (16.7%)	0.558
Cardiovascular Diseases	30 (9.25%)	8 (10.25%)	0 (0.0%)	0.728
Chronic Obstructive Pulmonary Disease	9 (2.8%)	1 (1.3%)	0 (0.0%)	0.696
Rheumatological Diseases	2 (0.65%)	1 (1.3%)	0 (0.0%)	0.809
Number of post-COVID symptoms, mean (SD)	3.0 (1.65)	2.9 (1.8)	3.8 (2.4)	0.324
Post-COVID symptoms, n (%)				
Fatigue	228 (70.4%)	50 (64.1%)	5 (83.3%)	0.768
Pain Symptoms	136 (41.9%)	28 (35.8%)	2 (33.3%)	0.819
Memory Loss	88 (27.2%)	22 (28.2%)	2 (33.3%)	0.950
Hair Loss	81 (25.0%)	23 (29.5%)	1 (16.7%)	0.709
Concentration Loss	36 (11.1%)	8 (10.25%)	3 (50.0%)	0.509
Cognitive Blunting—Brain Fog	39 (12.1%)	4 (5.1%)	2 (33.3%)	0.065
Dyspnoea	60 (18.5%)	19 (24.35%)	1 (16.7%)	0.571
Ocular Disorders	36 (11.1%)	7 (9.0%)	2 (33.3%)	0.224
Anosmia	34 (10.5%)	5 (6.4%)	0 (0.0%)	0.432
Skin Rashes	48 (14.8%)	8 (10.25%)	0 (0.0%)	0.409
Gastrointestinal Disorders	20 (6.2%)	8 (10.25%)	1 (16.7%)	0.323
Ageusia	21 (6.5%)	2 (2.5%)	0 (0.0%)	0.358
Days in hospital, mean (SD)	8.25 (8.0)	7.4 (6.3)	6.2 (4.0)	0.554

**Table 5:** Pre-infection data and post-COVID symptoms according to the IFITM3 *rs12252* polymorphism.

	A/A (n = 345)	A/G (n = 58)	G/G (n = 5)	*p*-Value
Age, mean (SD), years	59.7 (13.7)	53.5 (14.8)	48.6 (11.9)	0.002
Sex, female n (%)	161 (46.7%)	33 (56.9%)	4 (80.0%)	0.349
Weight, mean (SD), kg.	79.5 (17.0)	80.6 (15.7)	96.8 (25.9)	0.07
Height, mean (SD), cm.	167 (9.5)	166 (10.0)	165.5 (9.5)	0.202
Number of co-morbidities, mean (SD)	1.2 (0.9)	1.2 (1.0)	2.0 (1.2)	0.168
Medical co-morbidities, n (%)				
Hypertension	124 (35.9%)	16 (27.6%)	3 (60.0%)	0.389
Obesity	73 (21.2%)	21 (36.2%)	4 (80.0%)	0.003
Diabetes	38 (11.0%)	5 (5.8%)	0 (0.0%)	0.669
Asthma	33 (9.6%)	5 (5.8%)	0 (0.0%)	0.771
Cardiovascular Diseases	33 (9.6%)	5 (5.8%)	0 (0.0%)	0.771
Chronic Obstructive Pulmonary Disease	9 (2.6%)	1 (1.7%)	0 (0.0%)	0.868
Rheumatological Diseases	1 (0.3%)	2 (3.45%)	0 (0.0%)	0.338
Number of post-COVID symptoms, mean (SD)	2.9 (1.7)	3.4 (1.7)	3.2 (1.9)	0.204
Post-COVID symptoms, n (%)				
Fatigue	234(67.8%)	46 (79.3%)	3 (60.0%)	0.605
Pain Symptoms	138 (40.0%)	27 (46.5%)	2 (40.0%)	0.540
Memory Loss	94 (27.2%)	16 (27.6%)	2 (40.0%)	0.863
Hair Loss	88 (25.5%)	14 (24.1%)	3 (60.0%)	0.309
Concentration Loss	41 (11.9%)	6 (10.35%)	0 (0.0%)	0.709
Cognitive Blunting—Brain Fog	39 (11.3%)	6 (10.35%)	0 (0.0%)	0.740
Dyspnoea	60 (17.4%)	19 (32.8%)	3 (60.0%)	0.04
Ocular Disorders	38 (11.0%)	6 (10.35%)	1 (20.0%)	0.823
Anosmia	31 (9.9%)	8 (13.8%)	0 (0.0%)	0.430
Skin Rashes	47 (13.6%)	7 (12.1%)	2 (40.0%)	0.268
Gastrointestinal Disorders	27 (7.8%)	2 (3.5%)	0 (0.0%)	0.427
Ageusia	17 (4.9%)	6 (10.35%)	0 (0.0%)	0.238
Days in hospital, mean (SD)	8.0 (7.7)	8.4 (7.4)	8.2 (8.7)	0.941

## Data Availability

All data derived from this study are presented in the text.
